# Involvement of VNUT-exocytosis in transient receptor potential vanilloid 4-dependent ATP release from gastrointestinal epithelium

**DOI:** 10.1371/journal.pone.0206276

**Published:** 2018-10-26

**Authors:** Hiroshi Mihara, Kunitoshi Uchida, Schuichi Koizumi, Yoshinori Moriyama

**Affiliations:** 1 Center for Medical Education and Career Development, Graduate School of Medicine and Pharmaceutical Sciences, University of Toyama, Toyama, Japan; 2 Department of Gastroenterology, Graduate School of Medicine and Pharmaceutical Sciences, University of Toyama, Toyama, Japan; 3 Department of Physiological Science and Molecular Biology, Fukuoka Dental College, Fukuoka, Japan; 4 Department of Neuropharmacology, University of Yamanashi, Yamanashi, Japan; 5 Department of Membrane Biochemistry, Graduate School of Medicine, Dentistry and Pharmaceutical Sciences, Okayama University, Okayama, Japan; Indiana University School of Medicine, UNITED STATES

## Abstract

Adenosine triphosphate (ATP) modulates mechanosensitive vagal afferent nerves in the gastrointestinal tract. ATP is stored in secretory vesicles via the ATP transporter VNUT. Recently, the bisphosphate clodronate was reported to inhibit VNUT and was suggested to be a safe potent therapeutic option for chronic pain. Transient receptor potential vanilloid 4 (TRPV4) is activated by mechanical stimuli and some epoxyeicosatrienoic acids and becomes sensitized under inflammatory conditions. We have previously reported that TRPV4 and VNUT are expressed in mouse esophageal keratinocytes and that TRPV4 activation induces ATP release in gastric epithelial cells. Here we show the expression of TRPV4 and VNUT in normal human gastrointestinal cell derived cell lines (GES-1 and CCD 841) and in tissues from normal and VNUT-KO mice. TRPV4 agonists (GSK101 or 8,9-EET) induced an increase in cytosolic Ca^2+^ and/or current responses in mouse primary colonic epithelial cells and CCD 841 cells, but not in cells isolated from TRPV4-KO mice. TRPV4 agonists (GSK101 or 5.6-EET) also induced ATP release in GES-1 and CCD 841 cells, which could be blocked by the VNUT inhibitor, clodronate. Thus, VNUT inhibition with clodronate could represent a novel therapeutic option for visceral pain.

## Introduction

Purinergic signaling plays an important role in a variety of gut activities [1]. Neural ATP release is a co-transmitter in non-adrenergic, non-cholinergic inhibitory nerves involved in peristalsis and acts as a synaptic transmitter within ganglia. In contrast, epithelial ATP release in response to luminal distension has been proposed to act on ATP receptors in submucosal nerves to transduce signals to the CNS or enteric reflex; this hypothesis is based on the following observations [2]. Agonists of one of the ATP receptors, P2X3, stimulate mechanosensitive vagal afferent nerves in mouse stomach and esophagus. In the luminal fluid of the rat colon, the concentration of ATP is increased during distension, especially under inflammatory conditions [3, 4]. Moreover, P2X3-knockout (KO) mice display blunted responses to gastric distension [5]. ATP is stored in secretory vesicles by the vesicular nucleotide transporter, VNUT, and secreted via exocytosis upon stimulation. VNUT inhibitors identified thus far, however are toxic. Recently, clodronate, a bisphosphate, was reported to inhibit VNUT and is expected to be effective against chronic pain [6, 7].

Transient receptor potential channel vanilloid 4 (TRPV4) is a non-selective cation channel that is activated by mechanical stimuli, hypoosmolarity, heat or chemicals (GSK1016790A, 5,6- and 8,9-epoxyeicosatrienoic acid) and is sensitized by PAR-2, 5-HT and histamine [8, 9]. Recently it has been found that commensal bacteria-derived lipopolysaccharides also activate TRPV4 [10]. TRPV4 is present in diverse tissues including the colon [11], and we have reported that TRPV4 and VNUT are expressed in mouse esophageal keratinocytes and contribute ATP exocytosis [12]. Furthermore, TRPV4 activation induces ATP release in gastric epithelial cells [13] [14]. However, it is unknown whether TRPV4 activation induces ATP exocytosis from gastric and colonic epithelia. The main goal of this study was to establish whether clodronate could inhibit TRPV4 activation-induced ATP exocytosis in human gastrointestinal cells.

## Material and methods

### Animals

Male C57BL/6NCr (8-week-old; SLC), TRPV4-KO [15] and VNUT-KO mice [16] were used. All procedures involving the care and use of animals were approved by The Institutional Animal Care and Use Committee of the National Institutes of Natural Sciences and the University of Toyama and carried out in accordance with the National Institutes of Health Guide for the Care and Use of Laboratory Animals.

### Cell lines

The GES-1 gastric epithelial cell line (RRID:CVCL_EQ22) was obtained from the University of Texas at Austin. The GES-1 line was derived from a human nontumorigenic gastric mucosal epithelium and immortalized via SV40 [17]. GES-1 cells were maintained in RPMI supplemented with 10% fetal bovine serum, 1% glutamate, and 1% penicillin-streptomycin. The CCD 841 CoTr cell line (ATCC Cat# CRL-1807, RRID:CVCL_2872) was cultured in accordance with the manufacturer’s instructions. Cell lines were maintained in a humidified incubator at 33°C.

### Reverse transcription PCR analysis

Total RNA (1μg) was isolated using the RNeasy Mini Kit (Qiagen, Hilden, Germany). PCR was performed using FX neo (Toyobo, Japan) in an iCycler (Bio-Rad, CA, USA) with specific primer sets (Table 1). PCR conditions used for FX neo included: one cycle at 94°C for 2 minutes, 40 cycles at 98°C for 10 seconds, 55°C for 30 seconds, and 68°C for 90 seconds, followed by one cycle at 72°C for 2 minutes. Quantitative RT(qRT)-PCR was performed for mouse VNUT expression using the QuantiFast SYBR Green PCR Kit (QIAGEN) with the 7300 Real time PCR System (Applied Biosystems, CA, USA). Cycling conditions were 94°C for 5 minutes followed by 40 cycles of 94°C for 15 seconds and 60°C for 30 seconds. Data were collected and analyzed as values relative to GAPDH.

**Table 1:** Primer sequences for RT-PCR and qRT-PCR.

mTRPV4-F	ACAACACCCGAGAGAACACC
mTRPV4-R	CCCAAACTTACGCCACTTGT
mVNUT-F	GCCCTCTCTCAGGTTCAGTG
mVNUT-R	ACCTTGTTCTGGGGTCTGTG
mGAPDH-F	TGAAGGGTGGAGCCAAAAGG
mGAPDH-R	GGAAGAGTGGGAGTTGCTGTTG
hTRPV4-F	ACATTGTCAACTACCTGACGG
hTRPV4-R	ACAGGTAGGAGACCACGTTG
hVNUT-F	ACACACGAGCAGAGAGGAACACAA
hVNUT-R	TTTCTGGCTGTTGTCTGACTGGGA
hGAPDH-F	TGAAGGTCGGAGTCAACGGATTTGT
hGAPDH-R	CATGTGGGCCATGAGGTCCACCAC

### Immunochemistry

Immunochemistry was performed as previously described [12] using the antibodies summarized in Table 2. Cell lines and mouse tissues were fixed at 4°C for 6 hours. Tissues were placed in PBS–sucrose and embedded in OCT compound (Tissue-Tek, Elkhart, USA). Nonspecific antibody binding was reduced by incubation in BlockAce (Yukijirushi, Sapporo, Japan) for 1 hour at room temperature prior to antibody exposure. Preparations were analyzed using a confocal laser scanning microscope (LSM 760, Carl Zeiss, NY, USA). All experiments were repeated on specimens three times.

**Table 2:** Characteristics of primary and secondary antisera used for immunochemistry.

Tissue antigen	Host	Dilution	Source
VNUT	Rabbit	1:500	[34]
DAPI		1:1000	Dojin Chem
Secondary antibodies used for immunochemistry			
Antibody label		Dilution	Source
Goat anti-rabbit IgG-Alexa488		1:1500	Invitrogen, Inc.

### Isolation of primary mouse colonic epithelial cells

WT and TRPV4-KO mice were sacrificed by cervical dislocation. The colons were washed in cold (4°C) PBS (-) then incubated in trypsin solution (Invitrogen, CA, USA) at 4°C for 1 hr. Colonic epithelial cells were harvested and plated on Cell-Tak (BD Biosciences, NJ, USA)-coated glass cover slips and used for Ca^2+^-imaging and patch clamp experiments.

### Ca^2+^-imaging

Fura-2 fluorescence was measured in mouse primary colonic epithelial cells (n = 14 from three WT mice and n = 11 from three TRPV4-KO mice) and CCD 841 cells with a standard bath solution containing 140 mM NaCl, 5 mM KCl, 2 mM MgCl_2_, 2 mM CaCl_2_, 10 mM HEPES, and 10 mM glucose at pH 7.4 (adjusted with NaOH) at 25°C. Results are presented as ratios of fluorescence intensities obtained from fura-2 emissions at 340 nm and 380 nm. GSK101 (Merck KGaA, Darmstadt, Germany), 8,9-EET methylestel (Cayman Chemical, Michigan, USA) [18], ionomycin and RN1734 (from Tocris Bioscience, Bristol, UK) [19] were used as TRPV4 agonists, a positive control and a TRPV4 antagonist, respectively. Clodronate-liposomes (Katayama Chemical, Japan, 1 μM) were used to inhibit VNUT [20, 21]. F340/F380 and dose-response curves were calculated and acquired with an image processing system (IP-Lab, Scanalytics Inc., Rockville, MD) and ImageJ software (http://rsb.info.nih.gov/ij/). Changes in ratios (Δ) were calculated by subtracting the mean basal values from peak values.

### Electrophysiology

The standard bath solution was the same as that used in the Ca^2+^-imaging experiments. Pipette solutions for whole-cell recordings contained 140 mM KCl, 5 mM EGTA and 10 mM HEPES, at pH 7.4. Whole-cell recordings were collected from primary colonic epithelial cells three hours after isolation and were sampled at 10 kHz and filtered at 5 kHz for analysis (Axon 200B amplifier with pCLAMP software, Molecular Devices, CA, USA). Voltage ramp-pulses from 100 mV to +100 mV (500 ms) were applied every 5 seconds to generate an I-V curve. We used the ratio of the peak amplitude at negative (-80 mV) and positive (+80 mV) potentials as the rectification index.

### Measurement of ATP release (luciferin–luciferase assay)

ATP concentrations released from GES-1 and CCD 841 cells cultured in 12-well plates were measured using a luciferin-luciferase assay (ATP Bioluminescence Assay Kit CLS II, Roche Diagnostics, Basel, Switzerland) and a luminometer (Lumat LB 9507, Berthold Technologies, Japan) with slight modification to previously described methods [12, 14]. Cells cultured to 70–80% confluence and incubated in 500 μL bath solution for 30 min at room temperature (25°C) were used to measure basal ATP release. The superfusate was collected and gently replaced with another 500 μl of bath solution with or without the TRPV4 agonist, GSK101 (300 nM) or another endogenous TRPV4 agonist, 5,6-EET methylestel (Cayman Chemical, 1.5 μM) [18]. Half-normal saline was used as a positive control. The superfusate was collected after 15 min and the ratio of released ATP (15 min stimulation/30 min basal conditions) was calculated. An aliquot (200 μl) of superfusate was then mixed with 100 μl luciferin-luciferase reagent for luminometric ATP measurements. To block VNUT, cells were pre-treated with the specific VNUT inhibitor, clodronate (Merck KGaA, 1 μM) for 30 min [7]. Clodronate-liposomes were also used to inhibit VNUT.

### Data analysis and statistics

Values for qRT-PCR, Ca^2+^-imaging, patch-clamp experiments and ATP measurements are presented as the mean ± SEM from three or more independent experiments. A Student’s t-test was used to determine significance, set as *p* < 0.05.

## Results

### VNUT and TRPV4 expression in gastrointestinal epithelia

TRPV4 mRNA and protein have been shown to be expressed in the esophagus, stomach, intestine and colon [11–13, 22], and functional analysis of TRPV4 (Ca^2+^-imaging, electrophysiology and ATP measurement) has been performed in the esophageal and gastric epithelia [12, 13]. In the present study, we sought to examine VNUT expression throughout the gastrointestinal epithelium and performed functional analysis of TRPV4 in colonic epithelia.

**Fig 1:**
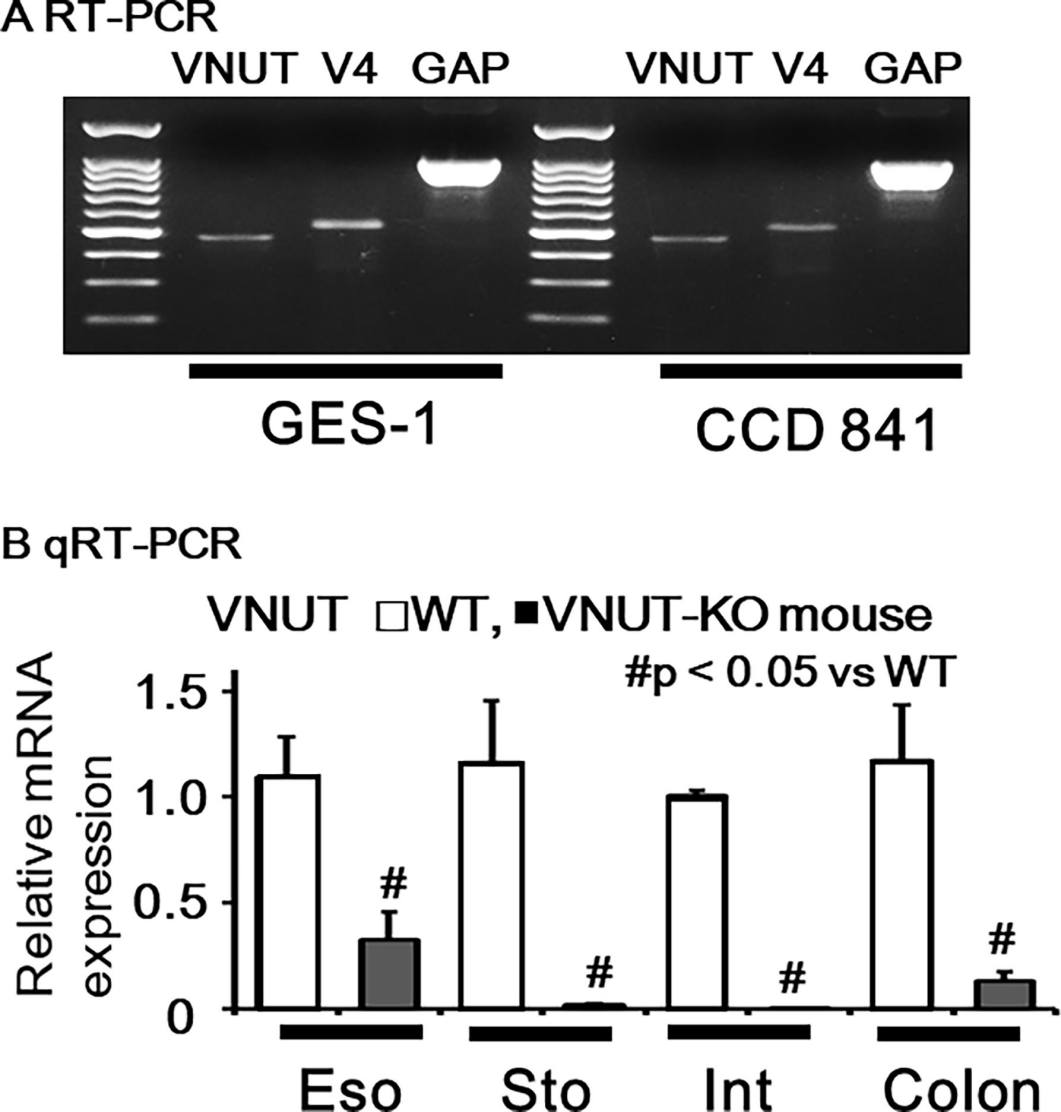
VNUT and/or TRPV4 mRNA expression in human cell lines and mouse tissues. (A) VNUT, TRPV4 (V4) and GAPDH (GAP) mRNA was detected in human gastric (GES-1) and colonic (CCD 841) epithelial cell lines. (B) Quantitative RT- PCR (qRT- PCR) indicated significantly lower mRNA expression in VNUT-knockout (KO) tissues (Eso; esophagus, Sto; stomach, Int; intestine, Colo; colon) compared to wild-type (WT) tissues (# P < 0.05 vs WT).

**Fig 2:**
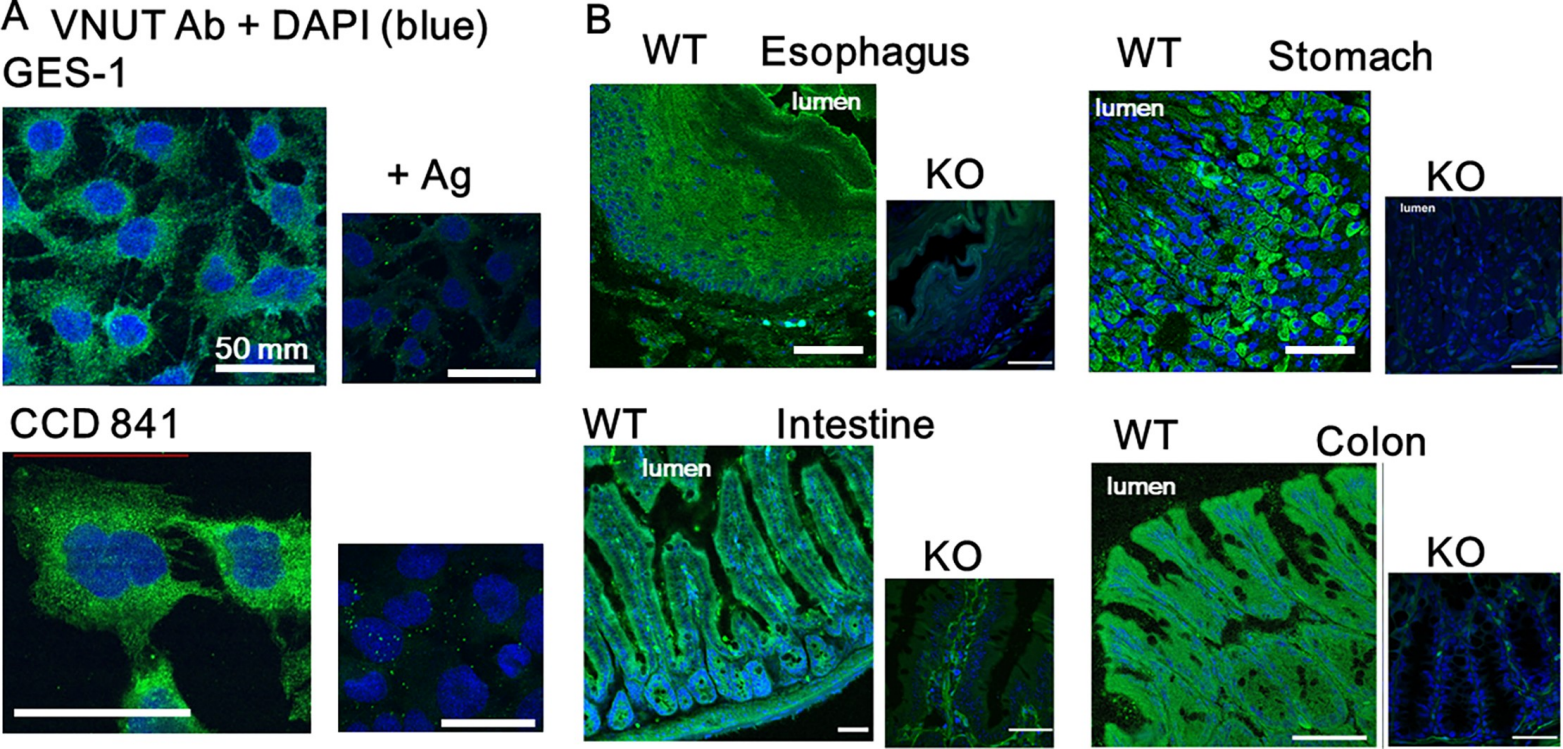
VNUT protein expression in human cell lines and mouse tissues. (A) VNUT expression was observed in GES-1 and CCD 841 cell lines. The punctate immunoreactivity was diminished with antigenic peptides (+Ag). (B) VNUT expression was observed in WT tissues (esophagus, stomach, intestine and colon) but not in VNUT-KO tissues. Scale bars indicate 50 μm. DAPI indicates cell nuclei.

VNUT and TRPV4 mRNA was detected in human gastric (GES-1) and colonic (CCD 841) epithelial cell lines (Fig 1A). Quantitative qRT-PCR identified VNUT mRNA in WT mouse tissues and significantly lower expression in VNUT-KO tissues (Fig 1B). We also examined VNUT protein expression in human gastrointestinal cell lines and mouse tissues. Punctate immunoreactivity was observed in GES-1 and CCD 841 cell lines and this reaction could be diminished with antigenic peptides, demonstrating that these reactions were specific to VNUT (Fig 2A). Strong homogenous immunofluorescent signal was confined to the epithelial cell layer in WT mouse tissues but not in VNUT-KO tissues (Fig 2B), demonstrating the ubiquitous expression of VNUT in the gastrointestinal epithelium and the specificity for VNUT.

### TRPV4-mediated increase in cytosolic Ca^2+^ ([Ca^2+^]_i_) and/or current responses in mouse primary colonic epithelial cells and CCD 841 cells

**Fig 3:**
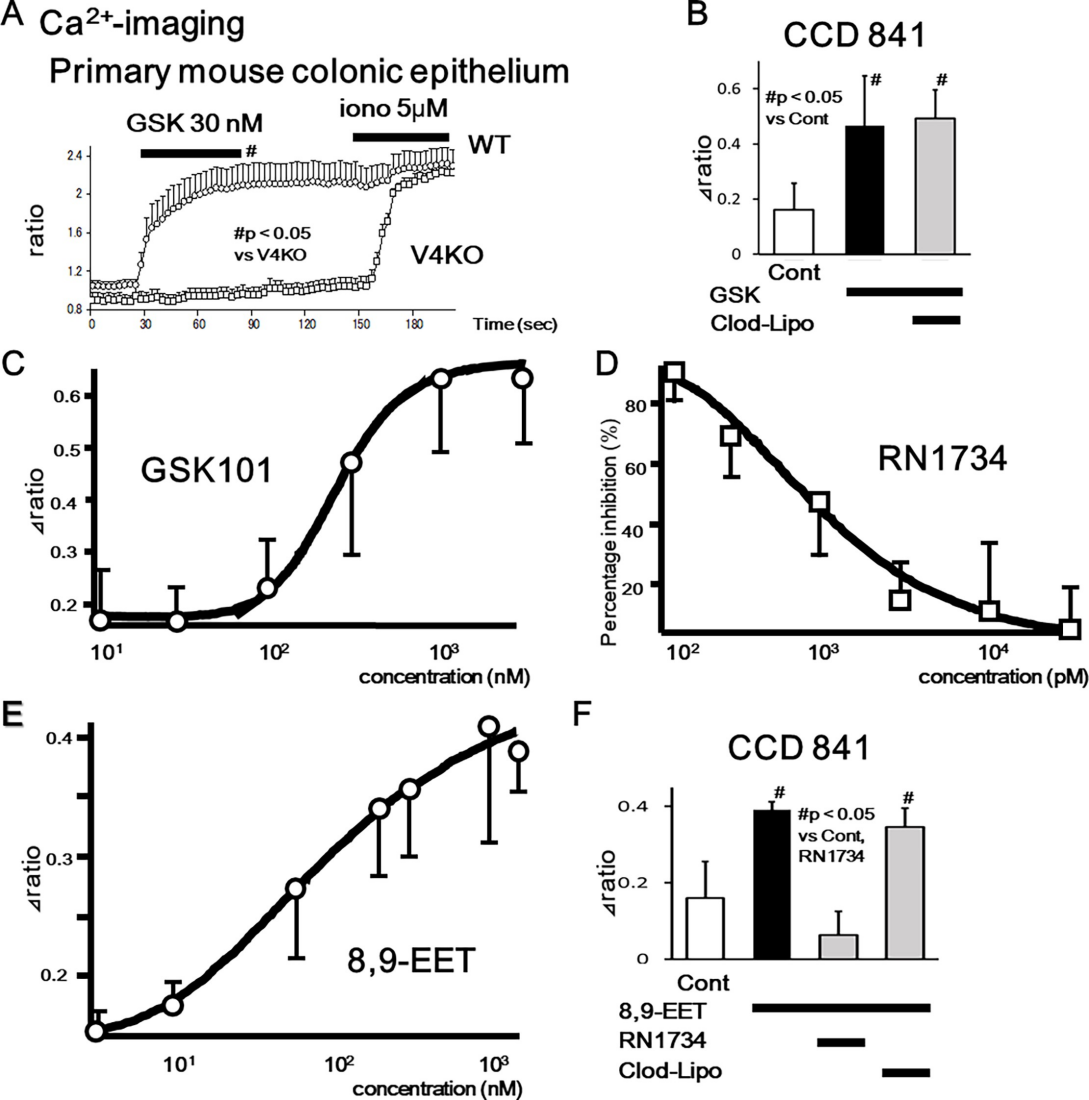
TRPV4-mediated increase in cytosolic Ca^2+^ ([Ca^2+^]_i_) in mouse primary colonic epithelial cells and CCD 841 cells. (A) [Ca^2+^]_i_ changes in response to the TRPV4 specific agonist, GSK101 (GSK, 30 nM), in WT or TRPV4-KO (V4KO) primary colonic epithelial cells (mean ± SEM). Ionomycin (iono) was used as a positive control. Bars indicate the period of chemical application. Significant increases in [Ca^2+^]_i_ at 90 seconds were observed in WT than V4KO (p<0.05). (B) GSK101 (300 nM) significantly increased [Ca^2+^]_i_ in CCD 841 cells (mean ± SEM, n = 17) compared to basal conditions (# p < 0.05 vs control). Clodronate-liposomes did not affect the [Ca^2+^]_i_ responses. (C) Dose-response [Ca^2+^]_i_ increase curve in CCD 841 cells responding to GSK101 determined EC50 value of 231.3 nM. (D) Dose-response [Ca^2+^]_i_ inhibition curve with a TRPV4 specific inhibitor, RN1734 in CCD 841 cells responding to GSK101 (3 μM) determined IC50 value of 702.6 pM. (E) Dose-response [Ca^2+^]_i_ increase curve in CCD 841 cells responding to an endogenous TRPV4 agonist, 8,9-EET determined EC50 value of 111.1 nM. (F) 8,9-EET (1.5 μM) significantly increased [Ca^2+^]_i_ in CCD 841 cells (mean ± SEM, n = 11) compared to basal conditions (# p < 0.05 vs control). RN1734 (10 μM) significantly inhibited the [Ca^2+^]_i_ responses, but clodronate-liposomes did not.

We had previously confirmed functional TRPV4 expression in esophageal and gastric epithelia using primary mouse cells and the rat gastric epithelial cell line, RGE01-1 [12, 13]. Therefore, to confirm functional TRPV4 expression in primary colonic epithelial cells and the human colonic epithelial cell line, CCD 841, we examined cell responses to the specific TRPV4 agonist, GSK101 and an endogenous TRPV4 agonist, 8,9-EET [18], using a fluorescent Ca^2+^-imaging system (10 μM, fura-2/AM). Response traces of [Ca^2+^]_i_ for WT and TRPV4-KO colonic epithelial cells in the presence of GSK101 (30 nM) showed that almost all cells isolated from WT colon responded to GSK101, indicating significant increases at 90 seconds in WT than in TRPV4-KO cells (p<0.05) (Fig 3A). This finding suggests that the majority of colonic epithelial cells express TRPV4 and that [Ca^2+^]_i_ responses to GSK101 were TRPV4 specific. Although human TRPV4 (EC50 = 5 nM) has a similar sensitivity to GSK101 compared to mouse (EC50 = 18.5 nM) or rat (EC50 = 10 nM) [18], the requirement for a higher concentration of GSK101 (300 nM) in order to increase [Ca^2+^]_i_ in CCD 841 cells (mean ± SEM, n = 17) compared to basal conditions (# p < 0.05 vs control) might be attributable to the lower expression level in CCD 841 cells (Fig 3B). Since clodronate-liposomes did not inhibit GSK101-induced [Ca^2+^]_i_ responses, clodronate had no inhibitory effect on TRPV4 channel activity. We next characterized TRPV4 functions in CCD 841 cells with dose-response curves, indicating EC50 value for GSK101 of 231.3 nM and IC50 value for a TRPV4 specific inhibitor, RN1734 responding to GSK101 (3 μM) of 702.6 pM (Fig 3C and 3D). We further characterized TRPV4 functions using an endogenous TRPV4 agonist, 8,9-EET, indicating EC50 value of 111.1 nM (Fig 3E). Since 8,9-EET (1.5 μM)–induced [Ca^2+^]_i_ increases was inhibited by a TRPV4 specific inhibitor, RN1734 (Fig 3F), 8,9-EET-induced [Ca^2+^]_i_ increases were mediated by TRPV4 channel function.

**Fig 4:**
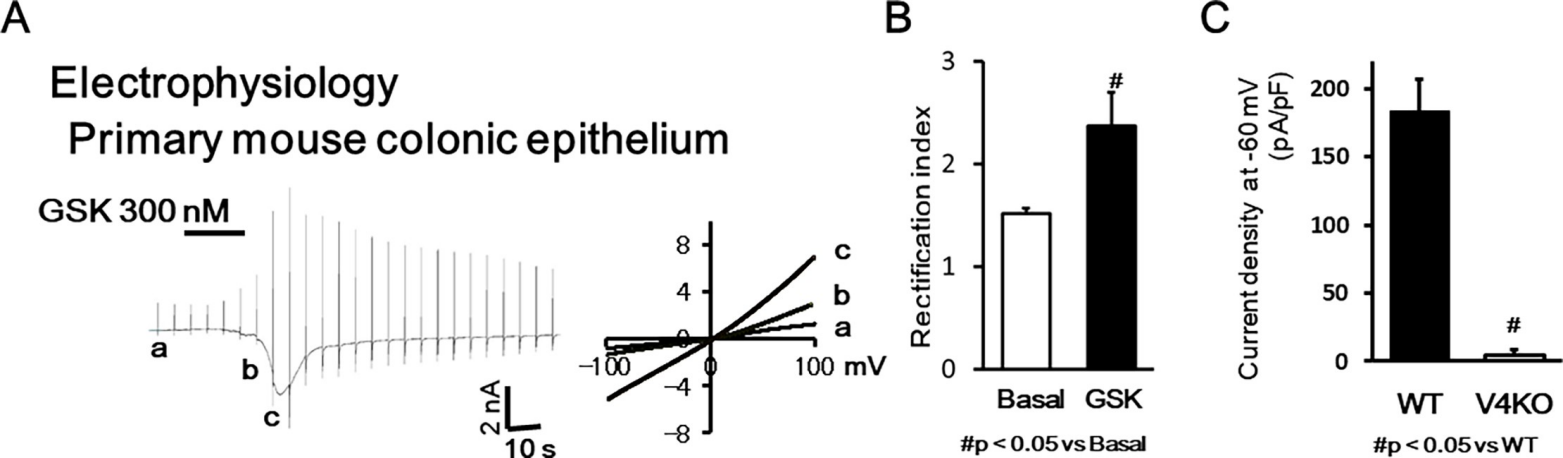
TRPV4-mediated current responses in mouse primary colonic epithelial cells. (A) TRPV4-mediated current responses in mouse primary colonic epithelial cells (mean ± SEM). GSK101 (300 nM) evoked inward current responses in WT primary colonic epithelial cells. Currents in response to ramp-pulses at points a, b and c (left) are shown (middle), with a strong outwardly rectifying current-voltage relationship (reversal potential of 0.4 mV). (B) Summary data of rectification between basal condition (at “a” in Fig 4A) and GSK101-induced currents (at “b” in Fig 4A) in WT cells (n = 5; p < 0.05 vs. Basal). (C) Significantly larger inward current densities (pA/pF) at -60 mV were obtained from WT cells (n = 5) than from TRPV4-KO cells (n = 5; # p < 0.05 vs. WT).

We next performed patch-clamp experiments with colonic epithelial cells isolated from mouse in the presence of GSK101 (300 nM) and observed inward current responses with an outwardly rectifying IV-relationship in WT but not TRPV4-KO cells (Fig 4A and 4B)[18]. Current responses were observed in all 5 trials with WT colonic epithelial cells but completely absent with TRPV4-KO cells, which suggests that the majority of colonic epithelial cells expressed TRPV4. GSK101 (300nM) evoked currents with an average current density of -183.5 pA/pF at -60 mV in WT cells. These values were similar to previous reports (Fig 4C)[23, 24]. These data strongly indicate the functional expression of TRPV4 in the colonic epithelium.

### VNUT-mediated ATP release induced by TRPV4 agonists in GES-1 and CCD 841 cells

We previously reported that a TRPV4 agonist induces ATP exocytosis in esophageal keratinocytes [12] and that TRPV4 agonists induced ATP release in the RGE1-01 rat gastric epithelial cell line [13]. To examine whether TRPV4 stimulation induces ATP release via VNUT-mediated exocytosis in human epithelial cells, we measured ATP release in GES-1 and CCD 841 cells using the luciferin-luciferase assay and a specific VNUT inhibitor, clodronate [7].

**Fig 5:**
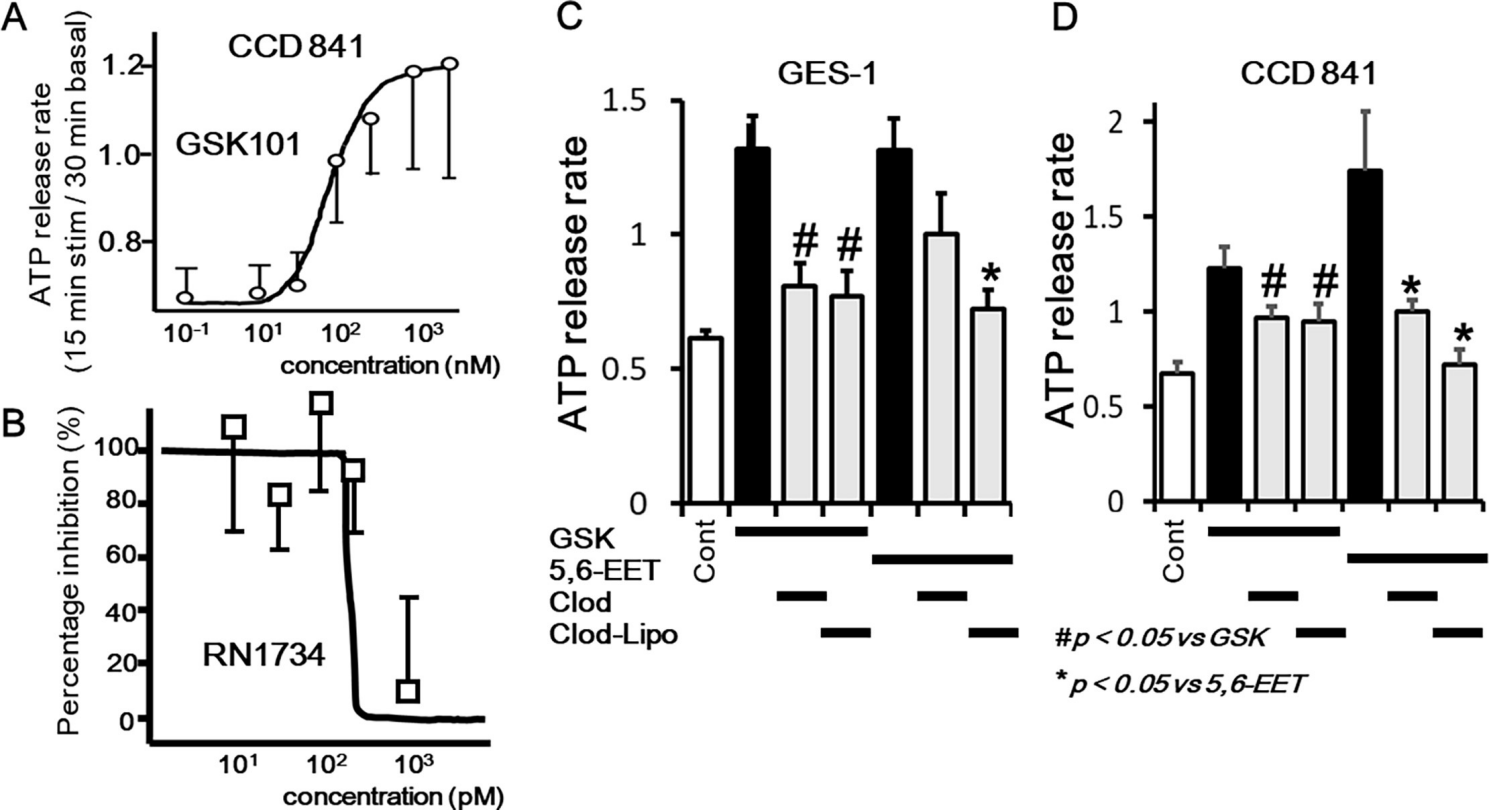
TRPV4 agonists-induced VNUT-mediated ATP release in CCD 841 and GES-1 cells. (A) Dose-response ATP release rate curve in CCD 841 cells responding to GSK101 determined EC50 value of 87.5 nM. (B)Dose-response ATP release rate inhibition curve with a TRPV4 specific inhibitor, RN1734 in CCD 841 cells responding to GSK101 (3 μM) determined IC50 value of 142.2 pM. (C, D) GSK101 (GSK, 300 nM)-induced ATP release in GES-1 and CCD 841　cells was significantly inhibited by pre-treatment with the specific VNUT inhibitor clodronate (Clod, 1 μM; p < 0.05). 5,6-EET (1.5 μM)-induced ATP release in GES-1 and CCD 841 cells was also significantly inhibited by pre-treatment with clodronate liposomes (Clod-Lipo, 1 μM; p < 0.05).

We firstly characterized dose-ATP release response and inhibition curves in CCD 841 cells, indicating EC50 value for GSK101 of 87.5 nM and IC50 value for a TRPV4 specific inhibitor, RN1734 responding to GSK101 (3 μM) of 142.2 pM (Fig 5A and 5B). Next pre-treatment with clodronate significantly inhibited TRPV4 agonist GSK101 (GSK, 300 nM)-induced ATP release (Fig 5C and 5D). The inhibitory effect of clodronate on another endogenous TRPV4 agonist, 5,6-EET in GES-1 cells did not reach statistical significance (p = 0.15). Given that clodronate likely shows reduced penetration of cell membranes, we next examined the inhibitory effect of clodronate-liposomes, which have improved cell permeability. Pre-treatment with clodronate-liposomes significantly inhibited 5,6-EET-induced ATP release in both GES-1 and CCD 841 cells (Fig 5C and 5D).

## Discussion

### Summary

The present study represents the first characterization of VNUT mRNA and protein expression in the mouse gastrointestinal tract and human gastrointestinal epithelial cell lines (GES-1 and CCD 841; Figs 1 and 2). The ubiquitous expression pattern throughout the gastrointestinal tract suggested that VNUT has a physiological function. Therefore, we confirmed the functional expression of TRPV4 in epithelial cells from mouse colon and CCD 841 cells (Figs 3 and 4). GSK101- and an endogenous TRPV4 agonist, 8,9-EET induced [Ca^2+^]_i_ responses were inhibited by RN1734, but not by clodronate. We demonstrated that ATP release induced by TRPV4 agonists (GSK101 and 5,6-EET) was inhibited by clodronate, a specific VNUT inhibitor [7], in GES-1 and CCD 841 cells (Fig 5), suggesting that TRPV4-induced ATP release from gastric and colonic epithelial cells is mediated by VNUT exocytosis.

### TRPV4 in the colonic epithelium

Few reports have focused on TRPV4 expression in the colonic epithelium. In the mouse and human colon, TRPV4 is localized to the epithelial cells and unidentified cells of the submucosal and muscular layers, and has been reported in the human colon cancer cell line, Caco-2 [11, 25]. TRPV4 agonists have been shown to increase intracellular calcium concentrations and chemokine release in human colon cancer cell lines and induced colitis in mice. The same group also reported that the level of 5,6-EET was increased in colonic biopsies obtained from patients with irritable bowel syndrome (IBS) [26]. Interestingly commensal bacteria-derived lipopolysaccharides also activates TRPV4 directly [10].The present study is the first report to identify functional TRPV4 expression in mouse colonic epithelial cells and to show that TRPV4-mediated ATP release in human colonic epithelial cells could be inhibited by clodronate. Although TRPV4 inhibition represents a potential therapeutic option for IBS or inflammatory bowel disease (IBD) [27], directly blocking TRPV4 might negatively affect normal physiological function [15].

### Purinergic receptor inhibition in the colon

The direct inhibition of purinergic receptors is another potential strategy for blocking purinergic signaling. Among the purinergic receptors, there is experimental evidence suggesting the efficacy of targeting A, P2X7, and P2X3 receptors for inflammatory pain, IBS and visceral pain [28]. The results from clinical-trials evaluating A1, A2A, P2X3 and P2X7 drugs for pain are pending. The main adverse events of P2X7 antagonist drugs are gastrointestinal (abdominal pain, nausea, diarrhea and vomiting), dizziness and headaches at the higher doses.

### Clodronate: An alternative purinergic inhibitory strategy

In 2008, the SLC17A9 gene was found to encode VNUT, which is responsible for the vesicular storage of ATP. VNUT-KO mice lose vesicular storage and release of ATP and exhibit resistance to the CFA model of acute inflammatory pain[7]. On the other hand, they are not lethal, and appear to be healthy[29]. It is thought that although VNUT is an essential component for vesicular ATP release in many cells, vesicular ATP release is not apparently necessary for maintaining life. Moreover, although the VNUT inhibitors identified thus far are toxic, clodronate has been used worldwide for the treatment of osteoporosis by intravenous or oral administration and has been shown to be analgesic in nature and safe without any severe adverse events including autonomic nervous system [30–32]. Therefore, VNUT inhibitors may be effective against pain [6]. Given that VNUT is expressed in various ATP-secreting cells including the esophagus [12], the identification of VNUT expression throughout the gastrointestinal epithelium suggested that VNUT inhibitors might also be effective against visceral pain. Moreover, intravenous injection of clodronate was shown to attenuate inflammatory pain by about 40% in mice [7]. The analgesic effect of clodronate was stronger than that of diclofenac and comparable to tramadol without any adverse effects. There are currently no clinical trials evaluating the use of clodronate for functional gastrointestinal disorders, visceral pain or IBD. Although as clodronate has difficulty permeating cell membranes, clodronate–liposomes were also evaluated in this study, clodronate-liposomes are used to deplete macrophages *in vivo* [33], and so there would be concerns about immune suppression. Clodronate-liposomes is restricted for studying VNUT *in vitro*.

## Conclusions

VNUT mRNA and protein were detected in human gastric and colonic epithelial cell lines and throughout the gastrointestinal epithelium of mice, and was shown to function as a transducer of TRPV4 agonists by inducing ATP release via exocytosis. Our results suggest that a specific VNUT inhibitor, clodronate, could represent a promising novel therapeutic drug for visceral pain and gastrointestinal inflammatory diseases without any severe adverse events.
